# A machine learning-based clinical decision support system for effective stratification of gestational diabetes mellitus and management through Ayurveda

**DOI:** 10.1016/j.jaim.2024.101051

**Published:** 2024-12-10

**Authors:** Nisha P. Shetty, Jayashree Shetty, Veeraj Hegde, Sneha Dattatray Dharne, Mamtha Kv

**Affiliations:** a Department of Information and Communication Technology, Manipal Institute of Technology, Manipal Academy of Higher Education, Manipal, India; b Department of Swasthavritta and Yoga, Yenepoya Ayurveda Medical College & Hospital, Kollarakodi, Naringana Village Bantwal, Manjanade, India; c Sri Dharmasthala Manjunatheshwara College of Ayurveda & Hospital, Kuthpady, Udupi, India

## Abstract

**Background:**

Gestational Diabetes Mellitus (GDM) is a metabolic condition that develops in course of pregnancy. The World Health Organization describes it as carbohydrate intolerance that causes hyperglycemia of varying severity and manifests itself or is first noticed during pregnancy. Early prediction is now possible, owing to the application of cutting-edge methods like machine learning.

**Objective:**

In the proposed empirical study, different machine-learning algorithms are applied to predict the prospective risk factors influencing the progression of GDM in gestating mothers.

**Materials and methods:**

The performance of these algorithms is evaluated through accuracy, precision, f1-score, etc. The lifestyle interventions and medications listed in Ayurveda literature are discussed for effective management of the disease.

**Results:**

Most of the proposed classifiers achieved a reasonable accuracy range of 75–82 %. Appropriate lifestyle changes, herbal remedies, decoctions, and churnas have all been shown to be useful in lowering the risk of GDM. Early detection using machine learning models can significantly reduce disease severity by facilitating timely Ayurvedic interventions.

**Conclusion:**

The proposed work is more focused on the identification of factors impacting GDM in expectant women. A balanced diet with physical exercise, proper medication, and better lifestyle management (through *Garbini Paricharya*) can control the perils of GDM if diagnosed prematurely.

## 1 Introduction

Diabetes is a significant public health issue characterized by excessive blood sugar levels. It occurs when the body is inept to properly use the insulin formed by the pancreas or when the pancreas is incapable of synthesizing insulin. It is the underlying factor in a number of related health conditions. *Diabetic peripheral neuropathy*, for example, is a type of nerve discomfort instigated by diabetes. Diabetes potentially increases eye issues, including *diabetic retinopathy* and *diabetic macular edema*. *Diabetic nephropathy*, kidney failure's root cause, is brought on by diabetes. Diabetes increases a person's risk of cardiac arrest. *Polycystic Ovary Syndrome (PCOS)*, a medical disorder that impairs the entire ovulation process, inflates the risk of developing diabetes. Diabetes can be categorized into three primary groups.

1) *Type 1* diabetes primarily affects children and teenagers. In this circumstance, the body either produces very little if any insulin.
2) Adults are more likely to have *type 2 diabetes* (90%). As a result of the body's improper response to insulin, blood glucose levels rise. Important risk factors for type 2 diabetes include obesity, a poor diet, high blood pressure, and inactivity.
3) *Gestational Diabetes Mellitus (GDM)* is characterized as glucose bigotry that develops or is diagnosed throughout pregnancy and normally resolves shortly after birth. Research has shown that as they age, women with GDM have a ten-fold greater risk of type 2 diabetes, a 2.5-fold increased risk of ischemic heart disease, and a two-fold increased risk of hypertension. Excess *amniotic fluid, ketoacidosis, preeclampsia, spontaneous abortion, stillbirth, and secondary infection* in pregnant women, as well as *foetal malformation hypoglycemia, hyperbilirubinemia, and hypocalcemia*, can all result from GDM. *Macrosomia*, or being significantly larger than average at birth, as well as *obesity and type 2 diabetes* are more probable in children exposed in utero [1].

Hence, if the liability can be predicted during the first trimester and prompt mediations are made, the effects of diabetes on pregnant women can be significantly reduced. An earlier study recommended using an *oral glucose tolerance test (OGTT diagnosis)* in the early stages of pregnancy for this purpose. However, intervening in every case repeatedly can be expensive and time-consuming. Therefore, along with the aid of conventional good practices that instruct the mothers to lead healthy lifestyles during pregnancy, a simple tool for assessing GDM should be introduced earlier in pregnancy to aid in assessing the hazards of GDM and identifying high-risk mothers warranting treatments, monitoring, and medication sooner.

Machine learning (ML) has recently gained popularity in a variety of preventative healthcare applications. Its advantages include high performance, resilience, generalizability, and reasonably priced computation. Machine Learning, or computer-assisted decision-making, assists humans by refining and analyzing intricate medical datasets to generate clinical observations. Such information mining from data is critical in the medical industry for early disease prediction and diagnosis. The increased access to medical data has driven efforts to construct multi-parametric machine-learning models for forecasting diverse medical disorders [2].

To accurately predict the likelihood that pregnant women will develop and experience gestational diabetes, it is crucial to identify the risk factors. The proposed system uses a set of significant influential aspects that have been carefully chosen through learning algorithms to identify and precisely envisage the possibility of gestational diabetes. Additionally, prompt control measures can significantly improve maternal and infant outcomes.

The interaction of the three *Doshas* and ten *Dushyas* (disturbed functioning of the principles that support the various bodily tissues) results in 20 subtypes of *Prameha*; some of these phenotypes have *sweet urine*, while others have urine that is colored differently, accentuating the inflammatory conditions linked to the metabolic syndrome. This malady is closely related to *Sthaulya* (i.e. obesity). *Type 1 diabetes* is correlated with *Sahaja Prameha, Jatah Pramehi, and Apathyanimittaja Prameha*, whereas *type 2 diabetes* is correlated with *Apathyanimittaja Prameha*. *Madhumeha* is a subtype of *Vataja Prameha* (Prameha with Vata predominance), which can evolve as type 1 diabetes starting in infancy or as the advanced stage of type 2 diabetes, requiring insulin. In *Charaka Samhita*, one of the classical Ayurvedic texts, the former is defined as *Jatah Pramehi Madhumehi* [3].

Though there is no direct mention of GDM, *Garba Vriddhi* is mentioned as a significant complication in pregnancy. *Garba Vriddhi* is characterized by an increase in abdominal size and perspiration. This is known as an *overweight fetus or Macrosomia*. A common protocol that most ayurvedic practitioners follow to inculcate a healthy pregnancy and delivery is *Garbini Paricharya*. Ayurveda focuses on changing Garbhini's lifestyle, which improves maternal health and foetal growth while reducing pregnancy complications. Pre-conception counseling, diet, herbal remedies, and yogasanas are beneficial as a supportive therapy when used in conjunction with conventional medicine under medical supervision [4,5].

### 1.1 Research goals and questions

Over the last decade, there has been a significant surge in publications concerning prediction models within obstetrics, demonstrating a notable rise in the desire to integrate machine learning and statistically derived methods into disease screening and detection [6]. The task at hand involves assessing the effectiveness of utilizing machine learning techniques on a selected set of predictor variables during the early stages of gestation to predict the occurrence of GDM. This paper aims to address the research question: "How effectively can machine learning models identify the most influential features for predicting Gestational Diabetes Mellitus (GDM) during early gestation, and which machine learning algorithm performs best in classifying GDM among gestating mothers?"

Considering the efficacy demonstrated by certain machine learning methods in identifying and forecasting various diseases, this study endeavors to address the matter by pursuing the following objectives.

• Compare the accuracy of various machine learning models to categorize pregnant women as GDM positive or GDM negative to improve risk-based screening for developing GDM.
• Employ a feature selection method to remove the “*curse of dimensionality*” problem and to find the most contributing features for effective evaluation. Problems that occur while analysing data in high-dimensional spaces are referred to as the “curse of dimensionality” and frequently result in overfitting and reduced model performance. In medical applications, addressing this issue is critical since it aids in identifying the most significant characteristics, decreases noise, and improves model accuracy and interpretability. This is especially crucial when dealing with complicated medical data, since identifying the most critical elements can lead to more accurate diagnosis and treatment decisions.
• Find effective ayurvedic interventions which aid in better management of GDM and promote safe delivery.

The structure of the rest of the article is as follows: The “Related Works” section investigates earlier studies conducted in the relevant field. Section “Materials and Methods” introduces the data, modeling process, and methodologies devised to address the problem statement. Section “Results” describes our final models’ performance and pertinent study interpretations. The study findings are discussed in Section 5. The relevance of Ayurvedic interventions to manage Gestational Diabetes is documented in the section “Effective Intervention Strategies”. Section 7 concludes the study with additional research recommendations.

## 2 Related works

We thoroughly examined original research papers that used machine learning to forecast the risk of GDM. Table 1 provides an overview of the papers that were identified to be pertinent.

**Table 1:** Pertinent Literature in the domain.

Sl No.	Authors	Study Outcome	Study Limitations/Scope for Future Work
1.	Xing et al., 2024 [7]	A web server named GDMPredictor predicted a woman's risk of acquiring gestational diabetes mellitus (GDM) during the course of her pregnancy using state-of-the-art machine learning methods. It tailored therapy recommendations based on biochemical indications such *A1MG, BMG, CysC, CO2, TBA, FPG, and CREA*. After it was trained on a dataset of 3467 expecting moms, its effectiveness was evaluated using auPRC and AUC measures. The obtained AUC value was 0.967.	Due to data limitations, certain variables, including racial traits and family history, were left out of the model, which could have an impact on the prediction's accuracy. The data was gathered from a single source of population. For the suggested work, there is no outside validation.
2.	Kang et al., 2023 [8]	Most of the time and in most groups, the XGBoost algorithm outperformed the LGBM method in terms of performance. The model using the GDM risk variables that the American College of Obstetricians and Gynecologists (ACOG) suggested at baseline performed the worst across the board for both the batch and its sub batches. The model with variables generated by the Boruta method was chosen for clinical application even though the performance of the models using SHAP values and the Boruta algorithm was identical at each time point. This was because the model with the Boruta algorithm had fewer features than the model with SHAP values.	Due to certain tests not being run on all populations, there may be bias and missing data.
3.	Liao et al., 2022 [9]	The study compared least absolute shrinkage and selection operator (LASSO) regression and super learner, which contains classification and regression tree, LASSO regression, random forest, and extreme gradient boosting algorithms, to predict risks for pharmacologic treatment beyond MNT medical nutrition therapy. Tenfold cross-validated logistic regression based on super learner-selected predictors was used to develop a more straightforward and comprehensible model that included the timing of the diagnosis of GDM, the diagnostic fasting glucose value, and the status and frequency of glycemic control at fasting during the one-week post-diagnosis.	This study may have limitations because to the limited number of algorithms examined in the Super Learner (SL) library that was selected. Due to the possibility of more efficient adaptive and smooth learners than those now being studied, this limitation may restrict our capacity to fully optimize model performance.
4.	Du et al., 2022 [10]	For various feature combinations, SVM achieved the highest balanced accuracy of all models. AUC-ROC of 0.792 was achieved by the model which included all the features.	Using intelligent optimization algorithms can improve model performance yet further.
5.	Zhang et al., 2022 [11]	Four of the most employed features in the models produced by the different feature selection techniques were *Body Mass Index* (*BMI), fasting blood glucose, maternal age, and family history of diabetes*. The cumulative sensitivity and specificity of the ML models that predicted GDM were 0.69 and 0.75, respectively, resulting in a combined AUROC of 0.8492. The most popular machine learning technique, logistic regression, had an overall pooled AUROC of 0.8151, while non-logistic regression models did better, with a total pooled AUROC of 0.8891.	There is no set procedure for selecting features in medical research. Some studies took into account characteristics based on information from previous models along with prediction reliability, consistency, application, availability, and cost. In contrast, others favored features having a statistically significant association with GDM. Secondly, if the internal bias analysis of the model is disregarded, the model's performance may be overestimated.
6.	Ali et al., 2022 [12]	XGBoost had superior performance as indicated by its AUC of 0.77 in comparison to RF and GBM. The results of this study utilizing the XGBoost model and SHAP value indicated that the presence of *gravidity, body mass index, maternal age, and prior GDM diagnosis* are all significant variables in the diagnosis of GDM.	There are biases inherent in the self-reported epidemiological data used to anticipate GDM. Another major issue is the class imbalance, which results from the algorithm being biassed because there are less GDM instances than there are undiagnosed cases. Therefore, in order to balance all the classes in the future, data-balancing algorithms like SMOTE and GANS can be employed. The suggested XGBoost's prediction powers can further be increased by using more dependable optimization techniques, such as Bayesian particle swarm optimization.
7.	Wang et al., 2022 [13]	The authors aimed to develop GDM risk prediction models that may be extensively utilized in the first trimester using four distinct techniques: a logistic regression model, two machine learning models, and a score-scaled model generated from a meta-analysis of 42 papers. For the training and validation sets, the logistic regression model (seven variables) produced AUCs of 0.799 and 0.834, respectively. Using the decision tree (DT) and random forest (RF) algorithms, AUCs of 0.825 and 0.823 for the training set and 0.816 and 0.827 for the validation set, respectively, were found.	Data were collected at a big tertiary institution with a high incidence of GDM. Because the study was retrospective in nature, the investigators were unable to collect comprehensive data on activity and food, which might have an effect on gestational weight gain and basal metabolic rates.
8.	Jader et al., 2022 [14]	The findings indicate that combining KMeans clustering, the elbow method, Mahalanobis distance, and the ensemble technique enhanced prediction accuracy (92% for k = 3). The study discusses the significance of hyperparameters in improving the performance of machine learning methods.	The authors intend to expand on the proposed work to improve adaptive healthcare applications in areas where this issue has received insufficient attention.
9.	Shankar et al., 2021 [15]	Gradient Boosting received an accuracy score of 0.77, which was the highest of all the models included.	Future developments could add more real-time features, which would improve the study's findings.
10.	Ye et al., 2020 [16]	The Gradient Boosting Decision Tree model outperformed all others. GDM was strongly associated with fasting blood glucose, HbA1c, triglycerides, and BMI.	The data is from a single institution and has no external validation. The current study could contain bias. More prospective studies and studies on larger populations are needed to determine the apt features that could detect GDM at reduced costs preventing adverse pregnancy outcomes. Second, this retrospective study omitted information on socioeconomic status, diet, and physical activity, which have been implicated as risk factors for GDM.
11.	Qiu et al., 2017 [17]	A cost-sensitive hybrid model of logistic regression, support vector machine and CHAID tree achieved a specificity of 0.998	No new insights were discovered regarding the risk factors affecting GDM

## 3 Materials and Methods

**Fig. 1:**
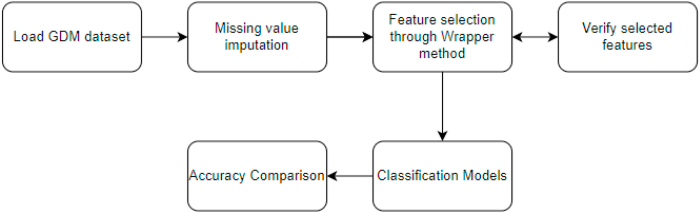
Proposed model.

Fig. 1 puts forth the steps followed in this research.

### 3.1 Data set

**Fig. 2:**
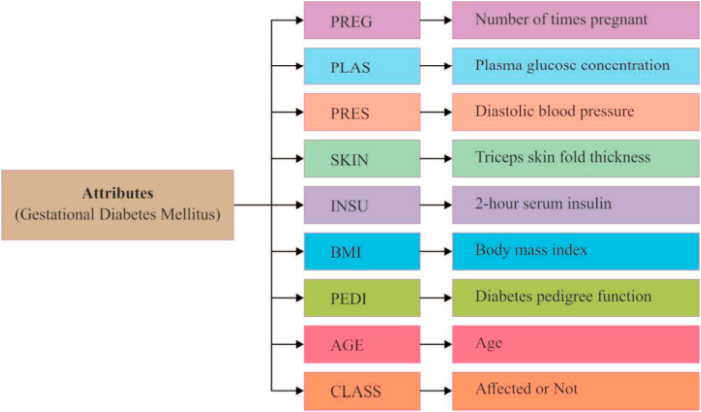
Features used in the study [15].

The study carried out by Nagaraj P et al. [18] served as inspiration for the selection of the data set. An overview of the attributes in the data set is provided in Fig. 2.

The dataset contains no null or missing values. However, based on domain expertise, the following attributes have inconsistent values: glucose concentration (Gluc), blood pressure (BP), skin fold thickness (Skin), insulin, and BMI, where zero values are not within the normal range and are thus erroneous. The process of impugning missing data involves substituting them with statistical estimates of their values. The purpose of any imputation technique is to generate a complete dataset for training machine learning models. Many investigations have indicated the imputation of real-time data sets using different techniques such as the mean, standard deviation, and a wide range of other statistical methods [19].

### 3.2 Feature importance and selection

The techniques used to evaluate the significance of each input feature for a specific model are termed “feature importance,” with the scores indicating the relative importance of each feature. Correlation measures the relationship between two characteristics, with significance assessed by a numerical value ranging from 0 to 1; positive values denote a direct correlation, while negative values indicate an inverse correlation. The extensive volume of data and complexity resulting from high dimensionality pose a challenge to the learning process, requiring the need for feature selection. In cases where numerous irrelevant features are present, some of which offer little value during learning, computational complexity, and overfitting issues tend to arise in learning models. This decreases learning accuracy. Although advantageous, feature selection necessitates more effort to produce an ideal subset that faithfully replicates the original dataset. Filter methods, wrapper methods, embedded methods, and hybrid methods are the different types of feature selection techniques used in classification. The proposed method employs the wrapper method for feature selection. Wrapper methods select features based on a specific machine learning algorithm tailored to a particular dataset. Employing a greedy search approach, they evaluate all possible feature combinations against a predefined criterion to determine the optimal set of features. They then choose the set of features that, when combined, produce the best outcomes for the given machine learning algorithm.

### 3.3 Modeling

In this study, we trained four different classifiers using K-nearest neighbor, random forest, support vector machine, and Naive Bayes, and then evaluated the classifiers on the data set. Furthermore, hyperparameter tuning is used to get the model working to its full potential. Finding a set of optimal hyperparameter values for a learning algorithm is what hyperparameter tuning entails. By curtailing a predetermined loss function, the chosen set of hyperparameters augments the model's performance and yields better results with fewer errors [20,21].

### 3.4 Ensemble

The fundamental tenet of the ensemble model [22] is that weak learners can be combined to create stronger learners. In addition to the base classification algorithms (mentioned above), the current study also employs the ensemble techniques for GDM prediction listed below.

• Stacking: Stacking is an ensemble learning technique that uses a meta-classifier or a meta-regressor to combine multiple classification or regression models. Following the training of the base-level models on the entire training set, the meta-model is trained using the features produced by the base-level models.
• Weighted Averaging: Model averaging is a method of ensemble learning where the contribution of each member to the final prediction is equal. While some models are known to perform notably better than others, this method requires that all ensemble members be equally skilled. An improvement over a model-averaging ensemble is a weighted ensemble, where each member's contribution to the outcome is weighed according to the model's individual performance. The weights of each model can be used to represent the proportion of confidence in each model as they're all small positive values that add up to one.
• Majority Voting: To predict class labels, the Voting Classifier amalgamates disparate machine learning classifiers with distinct conceptual approaches and employs a majority vote (hard vote) or an average predicted probability (soft vote). In this case, the ensemble output is based on the prediction with the most votes.
• Bagging: Decision trees are highly dependent on the data on which they are trained. The resulting decision tree, and consequently the predictions, can differ significantly if the training data is altered (for example, if a tree is trained on a subset of the training data). To reduce such observed high variance, bagging (as illustrated in the steps below) is incorporated.
  ○ Make numerous (for instance, 100) arbitrary replacement subsamples from the dataset.
  ○ On each sample, train a CART model.
  ○ For a new dataset, average all weak learner trees' predictions.

## 4 Result

Accuracy, precision, recall, and F1-measure were among the metrics used to evaluate these classification models in order to identify the one that performed the best overall. The number of accurate predictions made by a model in relation to the overall number of predictions is measured by the accuracy score. Recall (also known as sensitivity) is the percentage of relevant instances that were actually retrieved, whereas precision (also known as positive predictive value) is the percentage of relevant instances among the retrieved instances. The weighted average of Precision and Recall is the F1 Score. Generally speaking, F1 is more favorable than accuracy, particularly if there is an unequal distribution of classes.

### 4.1 Hyperparameters for the models

To boost the classifiers to perform at their best the classifiers are tuned to hyperparameter settings as shown in Table 2.

**Table 2:** Hyperparameters for the best performance.

Model	Best Parameters from Grid Search CV
Logistic Regression	{'C': 10.0, 'penalty': 'l1', 'solver': 'liblinear'}
SVM	{ n_jobs = −1,n_estimators = 700, oob_score = True, max_features = 'sqrt'}
Random Forest	{ gamma = 0.0001, C = 10, kernel = 'rbf'}

### 4.2 Imputation methods

The suggested method employs 3 types of imputation techniques.

1. Simple Imputer with mean, median or most frequent value imputation
2. KNN imputer
3. Iterative Imputer

**Fig. 3:**
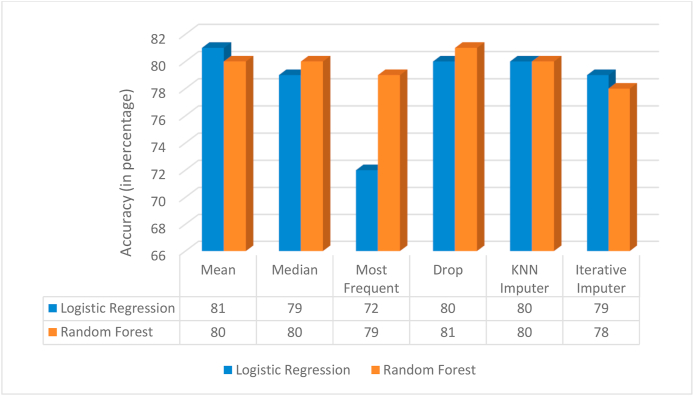
Comparison of accuracies of imputation methods.

The results obtained in Fig. 3 imply that for the chosen data set *mean method of imputation* performs well. Thus, this imputation method is used in the rest of the work. Although the *drop method* works the best it is not the most suitable approach owing to the loss in patterns caused by dropping the rows with missing values in them.

### 4.3 Feature importance

#### 4.3.1 Correlation

**Fig. 4:**
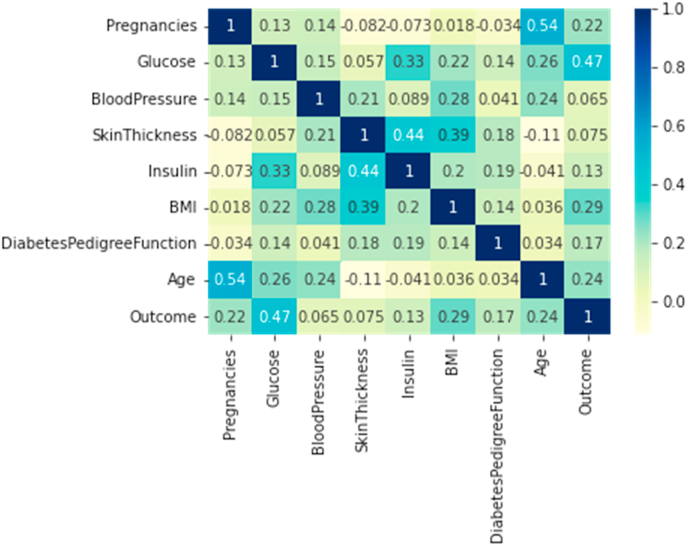
Correlation results.

The correlation statistical metric proves the most noteworthy features contributing to the outcome are ‘Glucose’, ‘BMI’, ‘Age’, ‘No. of Pregnancies’ (as shown in Fig. 4).

#### 4.3.2 Important features for mean imputation

**Fig. 5:**
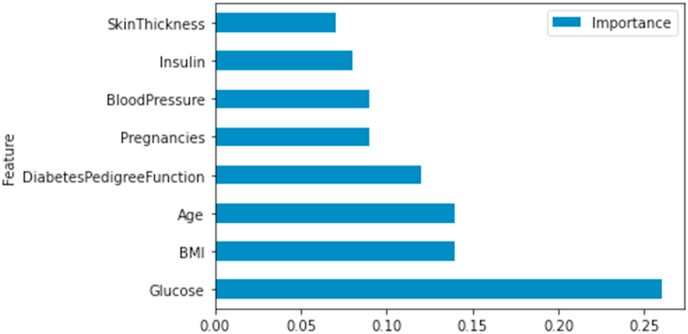
Feature ranking- random forest.

**Fig. 6:**
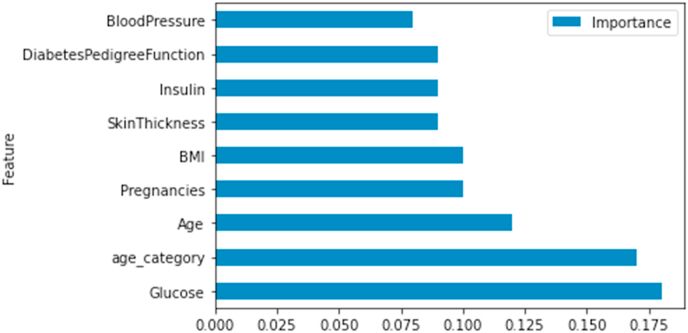
Feature ranking- XG boost.

Fig. 5, Fig. 6 validate the correlation results and show the ranking of the features according to Random Forest and XGBoost classifiers.

a. Random Forest_with hyperparameters_
b. XG Boost

### 4.4 Prediction accuracy (with all feature set)

**Fig. 7:**
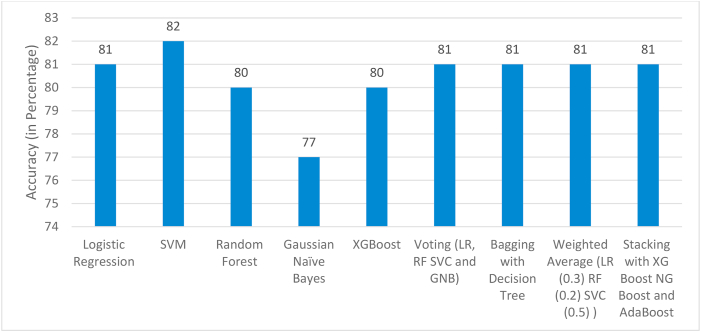
Accuracy Comparison – all features.

Fig. 7 and Table 3 puts forth the prediction results with all feature set included.

**Table 3:** Precision-Recall-F1 Score Comparison – all features.

Model	Precision for GDM positive	Precision for GDM negative	Recall for GDM positive	Recall for GDM negative	F1 Score for GDM positive	F1 Score for GDM negative
Logistic Regression _with hyperparameters_	0.87	0.8	0.4	0.97	0.55	0.88
SVM _with hyperparameters_	0.88	0.81	0.44	0.97	0.58	0.89
Random Forest _with hyperparameters_	0.69	0.84	0.56	0.9	0.62	0.87
Gaussian Naïve Bayes	0.64	0.8	0.44	0.9	0.52	0.85
XGBoost	0.71	0.83	0.53	0.91	0.71	0.83
Voting	0.41	0.97	0.87	0.8	0.55	0.88
Bagging with Decision Tree	0.76	0.82	0.5	0.94	0.6	0.88
Weighted Average	0.82	0.81	0.44	0.96	0.57	0.88
Stacking with XG Boost NG Boost and AdaBoost Level 1: XG Boost	0.56	0.91	0.72	0.84	0.63	0.87

### 4.5 Prediction accuracy (with wrapper method)

The effect of the Wrapper method on the subset of classifiers is shown in Table 4, Table 5.

**Table 4:** Accuracy Score Comparison – post Wrapper Method.

Model	No. of features	Top Features	Accuracy
Logistic Regression _with hyperparameters_	3	Glucose Age category BMI	0.83
	4	Glucose Age category BMI Diabetes Pedigree Function	0.79
SVM _with hyperparameters_	3	Glucose Age Pregnancy	0.8
	4	Glucose Age Pregnancy BMI	0.78
Random Forest _with hyperparameters_	3	Glucose BMI Age	0.8
	4	Glucose MI Age Insulin	0.79

**Table 5:** Accuracy Score Comparison – post Wrapper Method.

Model	No. of features	Top Features	Precision for GDM positive	Precision for GDM negative	Recall for GDM positive	Recall for GDM negative	F1 Score for GDM positive	F1 Score for GDM negative
Logistic Regression _with hyperparameters_	3	Glucose Age category BMI	0.84	0.83	0.5	0.96	0.63	0.89
	4	Glucose Age category BMI Diabetes Pedigree Function	0.74	0.8	0.4	0.94	0.55	0.87
SVM _with hyperparameters_	3	Glucose Age Pregnancy	0.92	0.79	0.34	0.99	0.5	0.88
	4	Glucose Age Pregnancy BMI	0.79	0.78	0.34	0.96	0.48	0.86
Random Forest _with hyperparameters_	3	Glucose BMI Age	0.71	0.83	0.53	0.91	0.61	0.87
	4	Glucose BMI Age Insulin	0.7	0.82	0.5	0.91	0.58	0.86

The obtained results show that with less no. of features (3–4) comparable accuracies are observed in all classifiers highlighting the significance of the chosen features and the efficacy of the algorithms.

## 5 Discussion

### 5.1 Correlation of the data set with ayurvedic principles

The concept of gestational diabetes in Ayurveda refers to a disruption in the equilibrium of doshas (*Pitta*, *Kapha*, and *Vata*), tissues (*Dhatus*), and waste products (*Malas*) during pregnancy, which results in less efficient utilization of glucose [24].

Several noteworthy factors are thought to have a role in the development of gestational diabetes, including [24].

• A dosha imbalance: According to Ayurveda, a dosha imbalance, especially between Vata and Kapha, is a contributing cause of gestational diabetes. A high Vata dosha can interfere with the pancreas' normal processes, resulting in insulin resistance and poor glucose management. Analogously, the channels (srotas) that allow the body to use and transport glucose can become blocked by an excess of Kapha dosha.
• Impaired Agni (digestive fire): Ayurveda places a strong focus on the necessity of a balanced Agni for healthy metabolism and digestion. Pregnancy-related Agni weakness or impairment can result in insufficient food digestion and absorption, which can cause ama (toxins) to build up and disrupt glucose metabolism.
• Ama Buildup: Per Ayurveda, the body's buildup of ama (toxins) can disrupt cellular metabolism, reduce insulin sensitivity, and heighten the risk of developing gestational diabetes. Poor food choices, impaired digestion, and lifestyle decisions that interfere with the body's natural detoxifying mechanisms can all lead to the accumulation of ama.
• Poor food and lifestyle choices: According to Ayurveda, unhealthy eating patterns and sedentary lifestyles are major causes of gestational diabetes. Overindulging in sugary, fatty, and heavy meals combined with insufficient exercise might worsen digestion, raise Kapha dosha, and cause pregnancy-related obesity, insulin resistance, and glucose intolerance.
• Stress and emotional factors: Ayurveda acknowledges the impact of mental and emotional aspects on physical health, including the prevention of gestational diabetes. Prolonged stress, worry, and emotional instability can cause hormonal imbalance, raise cortisol levels, and damage the neurological system, all of which can lead to insulin resistance and poor glucose management.
• Genetic predisposition: Ayurveda recognizes that a person's genetic makeup might predispose them to specific medical disorders, such as gestational diabetes. Particularly in women with a family history of diabetes or metabolic diseases, inherited imbalances in doshas, weak Agni, and other genetic predispositions may raise the risk of developing pregnant diabetes.

Table 6 examines the significance of the relevance of the attributes in the data set in contributing to GDM and their correlation with Ayurvedic principles.

**Table 6:** Correlation of the data set attributes with Ayurvedic principles.

Feature	Ayurvedic Perspective in correlation with GDM
Number of pregnancies	• Multiple pregnancies deplete “Ojas” (vital essence) and weaken “Dhatus” (body tissues) • Depletion of “Rasa Dhatu” (plasma) and “Rakta Dhatu” (blood) is considered key in developing “Garbhaja Prameha” (pregnancy-related diabetes)
Plasma glucose concentration	• Correlates with “Madhumeha Lakshana” (symptoms of diabetes) • Indicates imbalance in “Rasa Dhatu” and improper functioning of “Agni” (metabolic fire) • Relates to “Avila Mutrata” (turbid urine) due to excess “Kleda” (moisture)
Diastolic BP	• Indicates “Rakta Dushti” (vitiation of blood) and “Vata Prakopa” (aggravation of Vata dosha) • Linked to “Garbhaja Vata Vyadhi” (pregnancy-related Vata problems) • Can affect nutrition metabolism and circulation
Triceps skin fold thickness	• Refers to “Meda Dhatu Vriddhi” (excess adipose tissue) • Excess Meda can cause “Srotas Avarodha” (channel blockage) • May impair proper nutrition metabolism and contribute to insulin resistance
2-h serum insulin	• Indicates “Dhatvagni Mandya” (impoverished tissue metabolism) and imbalance in “Kleda” (body moisture) • Suggests disruption in the body's ability to handle glucose • Relates to improper “Ahara Pachankriya” (food digestion and metabolism)
BMI	• High BMI indicates “Sthoulya” (obesity) • Suggests excess of “Kapha Dosha” and “Meda Dhatu” • Considered critical to the pathophysiology of diabetes, especially GDM
Age	• Advanced maternal age associated with increased “Vata Dosha” • Can affect “Dhatu Parinama” (tissue transformation) and “Agni” (metabolic fire) • Vata predominance in older pregnant women is seen as a contributing factor to GDM
Diabetes Pedigree Function	• Aligns with the concept of “Kulaja Prameha” (hereditary diabetes) • Recognizes the role of “Beeja Dosha” (genetic factors) in disease development • Considered a strong indicator of susceptibility to GDM

### 5.2 Relevance of the features in ayurvedic assessments and correlation with Ayurveda *Nidan*

In Ayurvedic diagnosis of GDM, these aspects are evaluated as part of a comprehensive assessment.

- They contribute to determining the prevalent Dosha imbalance (*Vata*, *Pitta*, or *Kapha*) in GDM.
- They aid in determining the precise type of *Prameha* (among the 20 listed in traditional writings) that best describes the patient's situation.
- They help determine the stage of the sickness, whether it is in “*Purvarupa*” (prodromal) or full-blown **“***Rupa*” (symptomatic).
- They assist in understanding the “*Samprapti*” (pathogenesis) of GDM, allowing treatment techniques to be tailored more effectively.

Ayurvedic practitioners frequently use these indicators in conjunction with traditional diagnostic methods such as “*Nadi Pariksha*” (pulse examination), “*Jihva Pariksha*” (tongue examination), and “*Mutra* *Pariksha*” (urine examination) to form a complete picture of the patient's condition and determine the best treatment approach for GDM.

#### 5.2.1 Ayurvedic Nidan Correlation [25]

1. *Nidana* (Cause):
  - The dataset attributes help identify causative factors of GDM:
    ∗ Number of pregnancies: Multiple pregnancies can deplete Ojas and Dhatus
    ∗ BMI and skin fold thickness: Indicate Sthoulya (obesity), a key risk factor
    ∗ Age and family history: Point to Beeja Dosha (genetic predisposition)
  - These align with Ayurvedic concepts of *Nidana* such as *Ati-Sthoulya* (excessive obesity), *Avyayama* (lack of exercise), and *Kulaja* (hereditary factors).
2. *Purvarupa* (Prodromal symptoms):
  - Slightly elevated glucose levels or borderline BMI can indicate early stages of GDM
  - These correspond to Ayurvedic *Purvarupa* of *Prameha* like *Karapadadaha* (burning sensation in hands and feet) or Atitrisha (excessive thirst)
3. *Rupa* (Symptoms):
  - High glucose levels, elevated BP, and increased insulin levels represent full-blown symptoms
  - In Ayurveda, these align with Prabhuta Mutrata (excessive urination), Avila Mutrata (turbid urine), and Kshudhadhikya (excessive hunger)
4. *Samprapti* (Pathogenesis):
  - The combination of factors helps understand the disease progression
  - For instance, high BMI leading to elevated glucose represents the Ayurvedic concept of Meda Dhatu Vriddhi obstructing proper glucose metabolism

#### 5.2.2 Dosha imbalance determination

- *Kapha* predominance: Indicated by high BMI and skin fold thickness
  ∗ Relates to concepts like Sthoulya and Bahudrava Sleshma (increased bodily fluids)
- *Pitta* involvement: Seen in elevated glucose and BP
  ∗ Corresponds to Tikshna Agni (sharp digestive fire) leading to excessive hunger and thirst
- *Vata* influence: Reflected in age and family history
  ∗ Aligns with Vata's role in governing metabolic processes and genetic factors

#### 5.2.3 Prameha classification

- The dataset attributes help classify the type of Prameha:
  ∗ High BMI might indicate Medoja Prameha (diabetes due to fat tissue disorders)
  ∗ Elevated glucose could point to Kshaudrameha (honey-like urine)
  ∗ Family history might suggest Kulaja Prameha (hereditary diabetes)

### 5.3 Relevance of the chosen features in early identification of GDM

#### 5.3.1 Early prediction

In Ayurveda, the aforementioned variables—age, BMI, glucose levels, and diabetes pedigree function—are utilized to identify those who are at high risk of developing GDM. This makes it possible to take preventative measures before the illness worsens or develops. As an illustration.

• Elevated blood sugar levels, but not yet inside the diabetic range, may signify a prediabetic condition known as “Prameha Poorvaroopa” in Ayurveda (prediabetic symptoms).
• Age and BMI are important indicators of general health and metabolic efficiency, which Ayurveda links to the build-up of “Ama” (toxins) and “Agni” (digestive fire).
• Family history, or the diabetes pedigree function, corresponds with the Ayurvedic notion of “Beeja dosha,” signifying a hereditary susceptibility that necessitates caution.

#### 5.3.2 Influence on Mitigation [26]

1. Diet (*Ahara*):
  - For high BMI: Emphasis on light, easily digestible foods (laghu ahara) to reduce Kapha dosha.
  - For elevated glucose: Bitter and astringent tastes are recommended to balance sweetness in the body.
  - Age-specific recommendations: Younger women might receive different dietary advice compared to older women, based on their digestive strength.
2. Lifestyle (*Vihara*):
  - Age-appropriate exercise: Yoga asanas and pranayama techniques can be tailored according to age.
  - Sleep patterns: Recommendations can be offered for proper sleep hygiene based on age and body type.
  - Stress management: Techniques like meditation, tailored to individual prakriti and age can be recommended.
3. Herbs and formulations:
  - Blood glucose management: Herbs like Gymnema sylvestre (Gurmar) or Pterocarpus marsupium (Vijayasar) might be prescribed.
  - BMI-related: Formulations like Triphala or Trikatu for metabolism boosting in overweight individuals.
  - Age-specific: Rasayana (rejuvenating) herbs for older women to support overall health.
4. Panchakarma:
  - Detoxification procedures are recommended based on individual factors:
  - *Basti* (medicated enema) for Vata imbalance
  - *Virechana* (purgation) for Pitta imbalance
  - *Vamana* (therapeutic emesis) for Kapha imbalance
  - The intensity and frequency of these procedures would be adjusted based on age, BMI, and overall health status.

#### 5.3.3 Dosha assessment

The mentioned attributes help determine which dosha (Vata, Pitta, or Kapha) is predominantly imbalanced.

- High BMI often indicates Kapha imbalance
- Elevated glucose levels can suggest Kapha or Pitta imbalance
- Age can influence dosha status (e.g., advanced age may indicate increased Vata)

This assessment guides treatment strategies. For instance.

- *Kapha* imbalance might be addressed with more vigorous exercise and a lighter diet
- *Pitta* imbalance might require cooling herbs and stress reduction techniques
- *Vata* imbalance might be treated with grounding practices and nourishing foods

#### 5.3.4 Prakriti (constitution) analysis

Understanding an individual's innate constitution helps personalize the approach.

- *Vata* prakriti individuals might receive different dietary advice compared to Kapha prakriti individuals, even if their BMI is the same.
- *Pitta* prakriti individuals might be more prone to inflammation, requiring specific anti-inflammatory herbs.
- The susceptibility to stress and its impact on glucose levels might differ based on prakriti, influencing lifestyle recommendations.

#### 5.3.5 Synergy with ayurvedic diagnostic approach

The chosen key factors show a good synergy with the ayurvedic line of assessment. Glucose levels are used in conjunction with *Mutra Pareeksha* (urine examination) in Ayurvedic diagnosis. BMI is incorporated into *Pramana* (body measurements) and Samhanana (body constitution) assessment. Blood pressure is considered alongside *Nadi Pareeksha* (pulse examination). Insulin levels are interpreted in the context of *Agni* (digestive fire) and *Ama* (toxins) concepts in Ayurvedic medicine.

Thus, the major selected parameters contribute to determining the fundamental cause of imbalance in each individual, allowing for a more thorough and personalized strategy for anticipating and controlling GDM. While these characteristics aid in the early identification of GDM risk, they also guarantee holistic care that takes into account mental and physical elements, as well as preventive efforts customized to each patient's constitution and risk factors.

### 5.4 Linking the ayurvedic Mitigation approach with the machine learning diagnostic approach

The integration of Machine Learning (ML) with Ayurvedic principles offers a comprehensive approach to managing Gestational Diabetes Mellitus (GDM) risk during pregnancy [27]. ML algorithms predict GDM risk early using various health parameters, supporting the Ayurvedic *Nidan* process. High-risk ML predictions are followed up with traditional Ayurvedic diagnostic methods like *Nadi Pariksha* and *Ashtasthana Pariksha*. ML-identified risk factors are mapped to Ayurvedic concepts: for instance, high BMI correlates with *Kapha Vriddhi*, while elevated glucose indicates *Pitta Vriddhi*, aiding in explaining Samprapti (pathogenesis) from an Ayurvedic perspective.

For patients identified as high-risk by ML, monitoring focuses on early *Lakshana* (symptoms) like *Pipasa* (excessive thirst) and *Prabhuta Mutrata* (excessive urination). The *Garbhini Paricharya* (prenatal care) is tailored based on ML-predicted risk levels. Low-risk cases follow standard care, while moderate-risk cases emphasize *Pathya Ahara* (suitable diet) to balance identified *Doshas* and recommend specific pregnancy-safe Yoga asanas. High-risk cases involve intensified dietary interventions, such as incorporating more bitter and astringent tastes for high glucose risk and suggesting specific herbs like *Guduchi* or *Amalaki* based on the dominant *Dosha* imbalance.

Such approaches use ML risk profiles to inform the assessment of current Vikriti (imbalance) and customize interventions based on individual Prakriti. For ML-predicted high glucose risk, the focus is on Pitta-pacifying regimens, while high BMI risk emphasizes Kapha-reducing interventions. Periodic ML predictions assess the effectiveness of Ayurvedic interventions, guiding adjustments to Garbhini Paricharya practices throughout pregnancy.

This integration allows for earlier and more precise implementation of Ayurvedic interventions, more personalized care based on quantifiable risk factors, and a bridge between modern medical screening and traditional Ayurvedic practice. It enhances the explanation of GDM risk and progression in Ayurvedic terms and enables continuous refinement of care strategies throughout pregnancy. By respecting both the predictive power of ML and the holistic, personalized nature of Ayurvedic care, this approach creates a more comprehensive system for managing GDM risk, ultimately enhancing maternal and fetal well-being.

### 5.5 Translational value and implementation aspects of the proposed architecture: A medical personnel viewpoint

The machine learning (ML) model for predicting gestational diabetes mellitus (GDM) offers numerous advantages for medical personnel. It enables quick risk stratification, allowing for efficient resource allocation and personalized care plans. By identifying high-risk patients earlier than traditional methods, the model facilitates early intervention and implementation of preventive measures. In resource-constrained settings, it helps prioritize patients needing more intensive screening and follow-up [28,29]. The model's risk factor identification guides the tailoring of lifestyle interventions and monitoring strategies for individual patients. Unlike one-time tests, it allows for continuous risk assessment throughout pregnancy, enabling dynamic adjustment of care plans as new data becomes available. Integration with electronic health records (10.13039/100000081EHR) systems can provide real-time risk assessments, supporting clinical decision-making. Medical personnel can use the model's predictions as an educational tool to inform patients about their specific risk factors, potentially improving compliance with preventive measures. Compared to the oral glucose tolerance test (OGTT), the ML model offers several benefits: it provides non-invasive initial screening, potentially increasing compliance; enables continuous risk assessment throughout pregnancy; considers multiple risk factors for a more comprehensive analysis; can identify high-risk patients earlier in pregnancy; improves resource efficiency by determining who needs the more intensive OGTT; enhances patient comfort by avoiding fasting and glucose solution consumption; and can be applied at any prenatal visit. While the model does incorporate plasma glucose concentrations, it uses this as one of several factors, providing a more holistic risk assessment than glucose levels alone. It can utilize glucose values from routine prenatal blood tests and potentially show risk trends over time. The ML model can be used in conjunction with OGTT, helping identify who might benefit most from early or repeated testing. Its multifactorial approach, potential for earlier and ongoing risk assessment, and integration of routine clinical data provide significant advantages over relying solely on the OGTT. However, it's crucial to emphasize that the ML model should be viewed as a complementary tool to enhance, not replace, current clinical practices in GDM screening and management.

## 6 Effective Intervention Strategies

**Fig. 8:**
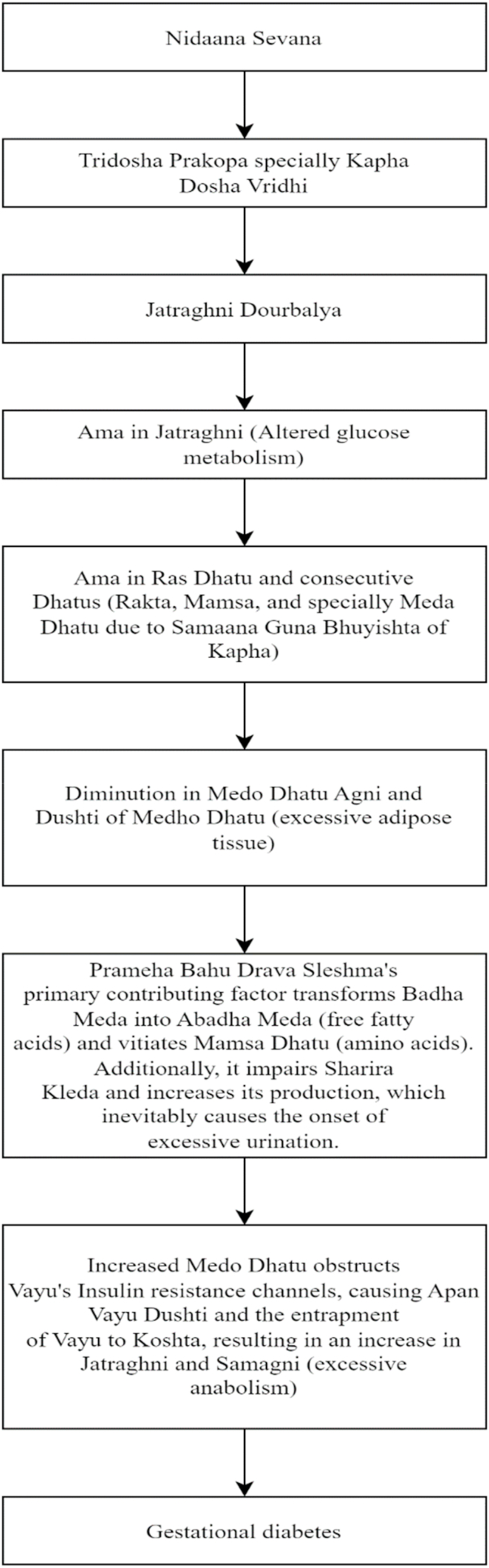
Disease pathophysiology in Ayurveda [30].

A brief glimpse of the disease pathophysiology in Ayurveda is illustrated in Fig. 8.

### 6.1 Ayurvedic understanding of Garbhini Prameha (GDM) [31]

*Garbhini Prameha*, often known as gestational diabetes, is caused by a variety of reasons according to Ayurveda. Pregnancy-related factors include increased *Kleda* (moisture) due to hormonal changes, *Vata* displacement by the expanding fetus altering *Agni* (digestive fire), and higher nutritional needs resulting in *Dhatu Kshaya* (tissue depletion). Pre-existing diseases such as *Kapha*-predominant *Prakriti*, *Prameha* in prior pregnancies, and pre-conception obesity all have a role. The illness is exacerbated by dietary factors such as eating irregular eating schedules and consuming large amounts of rich, sugary, and heavy meals. Lack of regular exercise and daytime sleep are two lifestyle variables that increase *Kapha*. Psychological issues such as pregnancy-related stress and sadness affect hormonal balance and metabolism.

The pathogenesis begins with increased *Kleda* and hormonal changes leading to *Dhatushaithilya* (tissue laxity) and *Vata* aggravation due to fetal growth. This affects Jatharagni (digestive fire), causing improper food digestion and *Ama* (toxin) accumulation in *Rasa Dhatu* (plasma). *Kapha* increases naturally during pregnancy, and combined with *Ama*, leads to *Srotorodha* (channel blockage). Pitta vitiation affects glucose metabolism. The condition primarily affects *Rasa*, *Meda*, and *Mamsa* tissues, with secondary effects on *Rakta* and *Majja*. It impacts *Rasavaha*, *Mutravaha*, and *Medovaha Srotas* (channels). The final manifestation involves *Ama-yukta Kapha* overflowing from the digestive tract to the periphery, with *Mutravaha Sroto Dushti* causing increased and altered urinary output.

Clinical features of *Garbhini Prameha* include excessive and turbid urination, increased appetite and thirst, lethargy, burning sensation in hands and feet, excessive sweating, weakness, body fatigue, and constipation due to *Vata* imbalance. These symptoms reflect the complex interplay of doshas, dhatus, and srotas in the Ayurvedic understanding of gestational diabetes [32].

#### 6.1.1 Factors making Garbhini Vulnerable to GDM and Upadravas/Udarkas of Garbhini Prameha [33]

*Garbhini Prameha* presents unique aspects that distinguish it from typical *Prameha*. The natural increase of Kapha during pregnancy complicates the usual pathogenesis, while Vata displacement due to fetal growth adds another dimension. *Agni* fluctuations throughout pregnancy stages affect disease progression. The need to maintain *Ojas* for both mother and fetus complicates treatment approaches. The sequential nourishment of fetal tissues adds complexity to the typical *Prameha Samprapti*. *Garbha*-specific effects include potential fetal macrosomia due to excessive nutrition and the risk of miscarriage in severe cases. These factors, combined with increased metabolic demands of the growing fetus, hormonal changes affecting *Agni* and *Dhatu Parinama*, *Ojas* depletion, *Apana Vayu* displacement, and excessive *Kapha* leading to *Srotorodha*, create a unique clinical picture that requires careful consideration in diagnosis and treatment.

The Ayurvedic perspective on *Garbhini Prameha* recognizes various potential outcomes, known as *Upadravas* or *Udarkas*, affecting both the mother and the fetus. Maternal outcomes may include *Garbhapata* (miscarriage) due to *Dhatu Kshaya* (tissue depletion), *Garbhasrava* (threatened abortion) from *Vata* imbalance, and *Rakta Dushti* leading to pregnancy-induced hypertension. Fetal outcomes can manifest as Sthoulya (macrosomia) due to excessive nutrition, Garbha *Vriddhihani* (intrauterine growth restriction) in severe cases, and *Vata Prakopa* in the newborn leading to hypoglycemia. Long-term consequences may involve *Sthayee Prameha* (persistent diabetes) post-delivery, *Kleda Vriddhi* predisposing to future *Prameha*, and *Dhatu Saithilya* increasing susceptibility to other disorders. This Ayurvedic understanding of GDM's outcomes highlights the complex interplay of *doshas*, *dhatus*, and *srotas* during pregnancy, offering unique insights that could complement modern medical approaches to GDM management.

#### 6.1.2 Benefits of early detection via machine learning for Garbhini Prameha management

The integration of Machine Learning (ML) for early detection in *Garbhini Prameha* management offers numerous benefits from an Ayurvedic perspective. ML algorithms can enhance Dosha assessment, provide *Ritu*-wise predictions, enable *Prakriti*-based risk stratification, and aid in early *Ama* detection. These technologies can also assist in evaluating Agni status, predicting Srotas function, estimating *Ojas* levels, and monitoring *Dhatu Parinama*. By leveraging ML for early detection, practitioners can offer customized *Pathya-Apathya* recommendations, guide Aushadha selection, and determine when to integrate modern medical interventions. Furthermore, ML models can potentially predict *Upadravas*, optimize *Garbha Vriddhi*, and inform post-partum care planning. This synergy between traditional Ayurvedic wisdom and cutting-edge technology enables a more proactive, personalized approach to *Garbhini Prameha* management, potentially improving outcomes for both mother and child by addressing the condition's unique aspects from an Ayurvedic standpoint.

### 6.2 Ayurvedic line of treatment when diagnosed with garbhini Madhumeha

#### 6.2.1 Mode of action of Ayurveda interventions in the management of GDM

Ayurvedic management of gestational diabetes mellitus (GDM) employs a comprehensive, multi-pronged approach targeting various aspects of metabolism and overall health. Dietary interventions play a crucial role by balancing *Agni* (digestive fire), which improves metabolism of carbohydrates, proteins, and fats, enhances digestive enzyme production, and optimizes nutrient absorption. These interventions also reduce *Ama* (toxins) formation, decreasing cellular insulin resistance and improving intracellular glucose utilization. Additionally, they pacify aggravated *doshas*: reducing excess body weight (*kapha*), regulating liver function (*pitta*), and stabilizing nervous system function affecting pancreatic activity (*vata*). Herbal medicines are utilized to improve insulin sensitivity by enhancing insulin receptor activity and increasing glucose transporter expression. They also enhance glucose uptake by cells through activation of the AMP-activated protein kinase pathway and regulate hepatic glucose production by inhibiting gluconeogenesis enzymes. *Panchakarma* therapies remove accumulated toxins, improve cellular metabolism, and enhance tissue responsiveness to hormones, including insulin. Yoga and exercise play a significant role by improving insulin sensitivity, reducing stress, and enhancing circulation and cellular metabolism. Specific practices like *Pranayama* (e.g., Bhramari, Anulom Vilom) activate the parasympathetic nervous system, potentially regulating blood glucose by reducing stress hormones. Stress management techniques, including meditation, reduce cortisol levels, improve insulin sensitivity, and balance the nervous system, optimizing hypothalamic-pituitary-adrenal axis function. Lifestyle modifications, such as early dinners and adequate sleep, align with natural circadian rhythms, improving insulin sensitivity and overall metabolic efficiency. Lastly, *rasayana* (rejuvenation) therapies strengthen *dhatus* (body tissues), enhancing cellular response to hormones and supporting proper tissue formation and function. This holistic approach addresses multiple facets of GDM, including insulin sensitivity, glucose metabolism, inflammation, oxidative stress, hormonal balance, and individual Dosha imbalances, providing a comprehensive strategy for managing the condition.

#### 6.2.2 Following Garbini Parichaya

Our Acharyas, were well aware that a developing fetus is influenced greatly by maternal psychic impressions in addition to factors like nutrition (*Ahara*) and environment (*Vihara*) [34] following certain regimen would aid in maintaining the *shareera Kledata*.

1. Dietary regimen

Eat a balanced diet, take *Deepana dravyas-treated Hridya drava, Madhura, and Snigdha* substances, Use the *Jeevaniya group of drugs* both externally and internally, use butter, ghee, and milk, and follow a diet that is appropriate for your location, season, and *Agni*. Make use of warm water.

Avoid excessive meat consumption, *atitarpana (excessively unctuous) and atikarshana (excessively emaciating) ahara*, dried, wet, putrid, and *vishtambi ahara*, as well as *tikshna, ushna, guru*, and irrational *aushadhas*. Also, avoid *madakaraka dravyas* (intoxicating substances) like wine and other alcoholic beverages.

2. Physical regimen

Avoid overexertion*, divaswapna, ratrijagarana, akala poorvakarma, panchakarma, raktamokshana, vega vidharana*, frequent outings, and visiting lonely places.

The Garbhini should thus adhere to a strict diet and behavior regimen during pregnancy. This regime is known as “***Garbhini Paricharya***.” [35,36].

Garbha Poshana: Garbha receives nutrition beginning at three months through *Upsneha and Upkleda*; however, once the placenta has formed, it receives nutrition directly from the placenta via *nabhinadi*. During the period of pregnancy, a complete diet constituting all the following *Vargas –Shali, Shimbi, Phala, Mool-Kanda,Dugdha, and Mamsa* is recommended by the Samhitas [37].

Monthly food regime suggested by Garbini Paricharya is listed in Table 7.

**Table 7:** Practices incorporated in Garbini Paricharya [38].

Month	Suggested Food	Scientific Significance
1	• Non medicated milk • Madhur, sheet, Liquid Diet • Madhuyashti, madhukapuspa with butter, honey and sweetened milk	• Supports a balanced diet when experiencing nausea and vomiting • Aids in preventing fluid loss from the body • Provides a cooling effect and helps stabilize the developing fetus • Madhura (sweet-tasting) substances possess growth-promoting properties
2	• Milk medicated with madhura rasa (sweet taste) drug	
3	• Milk with honey and ghrita	
4	• Milk with butter • Cooked sasti rice with curd, dainty and pleasant food mixed with milk, butter and Jangala mamsas	• Encourages the development of muscle tissue • Supports heart growth and function • Meat-based broths or soups help balance Vata dosha
5	• Ghrita prepared with butter extracted from milk • Cooked shastika rice with milk, Jangala mamsas along with dainty food mixed with milk and ghrita	• Supports brain development and cognitive functions • Enhances memory (Smrithi) and intellect (Buddi) • Aids in the formation of vital body tissues like blood (rakta), bone (ashti), and nervous tissue (Medhya) • Many pregnant women experience edema in feet by late second trimester due to kidney function changes • Gokhura, acting as a diuretic, may help prevent these kidney-related issues • Prithakpamyndi drugs contain both diuretics and anabolic agents, potentially addressing weight loss and kidney problems • Charaka recommends Yavagu in the 8th month for the mother's health and to promote a robust newborn • Asthapana and Anuvasana basti (types of enemas) may contribute to uncomplicated vaginal delivery (Nirupadrava prasava)
6	• Ghrita prepared from milk medicated with madhura (sweet) drug • Ghrita or rice gruel medicated with gokshura	
7	• Ghrita medicated with Prithakpamyndi group of drugs	
8	• Kshira Yawagu mixed with ghrita • Asthapanabasti with decoction of badari mixed with bala, atibala, satapuspa, patala etc., honey and ghrita	
9	• Asthapana is followed by Anuvasana basti • Cereals and Jangala mamsas until delivery	

#### 6.2.3 Specialized Garbhini Paricharya for GDM-high risk mothers

The Ayurvedic approach to managing high-risk pregnancies with Gestational Diabetes Mellitus (GDM) involves significant modifications to the conventional *Garbhini Paricharya* (prenatal care) guidelines. While traditional care emphasizes *Madhura* (sweet), *Snigdha* (unctuous), and *Sheeta* (cold) foods to promote fetal nourishment, the modified approach for GDM risk introduces a more balanced diet incorporating *Tikta* (bitter) and *Kashaya* (astringent) tastes, low glycemic index foods, and complex carbohydrates. The key differences lie in dietary recommendations, cooking methods, meal timing, exercise regimens, and herbal supplements. Unlike conventional care, which may limit physical activity, the modified approach encourages regular, moderate exercise and pregnancy-safe Yoga asanas. Herbal supplements focus on blood sugar balance and Agni stimulation, rather than general pregnancy tonics. Panchakarma therapies are adjusted to limit excessive oilation, and stress management techniques are emphasized to regulate cortisol levels. Sleep patterns are modified to discourage daytime napping, and a more frequent monitoring schedule is implemented to assess *Agni* status, *Kleda* levels, and overall *dosha* balance. These adaptations aim to balance fetal nourishment needs with the mother's metabolic challenges, offering a more personalized and effective management strategy for GDM while maintaining the core principles of Garbhini Paricharya.

### 6.3 Role and impact of yoga on pregnancies

Pregnancy causes unique hormonal shifts and stress in women. By decreasing blood flow and oxygen to the uterus or by triggering the placental stress system, which releases and circulates corticotrophin-releasing hormone, maternal stress is hypothesised to affect the intrauterine environment and modify foetal development during critical periods. The programming of the nervous system and the morphology of the developing brain in foetuses, children, and infants are all at risk due to maternal stress and stress-related peptide exposure during pregnancy. It's critical to manage maternal stress and provide pregnant moms with coping strategies for the unavoidable pressures and changes that come with pregnancy in order to enhance quality of life and support the health and development of newborns. Medication usage should be minimised during pregnancy due to the range of side effects and harmful consequences on the unborn as well as the mother.

Additionally, pregnancy hormones weaken ligaments, which can cause discomfort with joint and bone issues, especially those involving the pubic bone. Prenatal yoga focuses on respiration, stretches, and abdominal exercises that support the body's adaptation to pregnancy-related changes in the body as well as helping it get ready for labour.

Yoga is a safe and adaptable intervention that reduces stress in high-risk pregnancies. In *primigravida*, prenatal yoga has a significant impact on systolic blood pressure and foetal heart rates. Prenatal yoga has been demonstrated to increase IgA (an immune biomarker) and decrease cortisol (a stress hormone) during pregnancy. Cortisol levels, as well as inflammatory cytokines, interleukin (IL)-6, and tumour necrosis factor (TNF), have been shown to decrease following Yoga intermediation, while β-endorphin levels increase concurrently. As a potent modulator of the sympathetic nervous system during pregnancy, mindfulness meditation enhances parasympathetic functions in pregnant women. The following studies corroborate the findings of the effect of Yoga in high-risk pregnancies [[39], [40], [41], [42], [43]]. Table 8 presents effective asanas suitable during pregnancies.

**Table 8:** Yoga asanas recommended during pregnancy.

Trimester	Month	Recommended Asanas	Benefits
First (Weeks 1–12)	1–3	1. Tadasana (Mountain Pose)	Improves posture, strengthens spine, enhances circulation
		2. Vrikshasana (Tree Pose)	Improves balance, stability, strengthens legs, enhances concentration
Second (Weeks 13–26)	4–6	1. Baddha Konasana (Bound Angle Pose)	Opens hips, improves circulation, relieves fatigue
		2. Marjariasana (Cat-Cow Pose)	Strengthens spine, improves flexibility, enhances circulation
		3. Trikonasana (Triangle Pose)	Stimulates abdominal organs, improves digestion, reduces stress
Third (Weeks 27–40)	7–9	1. Setu Bandhasana (Bridge Pose)	Strengthens back, improves digestion, helps regulate blood sugar levels
		2. Shavasana (Corpse Pose)	Promotes relaxation, reduces stress, improves mental clarity
		3. Viparita Karani (Legs-Up-The-Wall Pose)	Opens hips, stretches inner thighs, aids in blood sugar management

### 6.4 Herbs in Ayurveda for effective GDM management [44]

Some of the most vital herbs are documented in Table 9.

**Table 9:** Effective herbs for GDM Management.

Sanskrit Name	Scientific Name	Ayurvedic Properties	Modern Pharmacology	GDM Implications
Triphala	*Emblica officinalis*, *Terminalia chebula*, *Terminalia bellirica*	Rasayana, balances doshas, detoxifying	Antioxidant, anti-inflammatory, hypoglycemic	Improves insulin resistance, manages blood glucose
Karavellaka	Momordica charantia L.	Pacifies Pitta and Kapha, manages blood sugar, improves digestion	Hypoglycemic, antidiabetic	Reduces blood glucose, improves glucose tolerance
Guduchi	Tinospora cordifolia	Rasayana, balances doshas, anti-inflammatory, antidiabetic	Hypoglycemic, antioxidant, anti-inflammatory	Controls blood glucose, improves insulin sensitivity
Amalaki	Phyllanthus emblica L.	Anti-diabetic, rejuvenative	Reduces postprandial glucose, enhances insulin secretion	Manages postprandial spikes, supports pancreatic function
Haridra	Curcuma longa L.	Anti-diabetic, anti-inflammatory	Improves insulin sensitivity, reduces oxidative stress	Improves insulin response, reduces inflammation
Jambu	Syzygium cumini	Anti-diabetic, absorbent	Alpha-glucosidase inhibitor, improves insulin secretion	Controls postprandial glucose, enhances insulin function
Methika	Trigonella foenum-graecum L.	Anti-diabetic, appetizer	Reduces fasting blood glucose, improves insulin sensitivity	Manages fasting glucose, improves insulin response
Meshashringi	Gymnema sylvestre	Destroys diabetes, reduces Kapha and fat	Reduces glucose absorption, increases insulin secretion	Controls glucose levels, supports pancreatic function
Ashwagandha	Withania somnifera	Rejuvenative, strengthening	Reduces glucose absorption, increases insulin secretion	Manages stress-induced hyperglycemia, supports metabolism

### 6.5 Etiopathogenesis of GDM [[45], [46], [47]]

Table 10 presents Etiopathogenesis of GDM with significant intervention strategies.

**Table 10:** Etiopathogenesis of GDM.

Feature	Characteristics	Intervention strategies
Insulin Resistance	• Pregnancy hormones increase insulin resistance • Resistance intensifies in the second and third trimesters	• Lifestyle modifications: Regular physical activity, balanced diet • Pharmacological interventions: Metformin in select cases
β-cell Dysfunction	• β-cells fail to adequately increase insulin production • Leads to hyperglycemia	• Lifestyle modifications: Regular physical activity, balanced diet • Pharmacological interventions: Metformin in select cases
Placental Factors	• Placenta produces hormones contributing to insulin resistance • Placental dysfunction may exacerbate GDM risk	• Close monitoring of placental health through regular ultrasounds • Optimizing maternal nutrition to support placental function
Genetic Predisposition	• Certain genetic polymorphisms associated with increased GDM risk • Family history of diabetes is a significant risk factor	• Early and more frequent screening for those with high genetic risk • Personalized preventive strategies based on genetic profile
Environmental and Lifestyle Factors	• Pre-pregnancy obesity • Advanced maternal age • Poor diet and lack of physical activity	• Weight management programs • Nutritional counseling • Stress management techniques • Regular blood glucose self-monitoring • Insulin therapy when lifestyle modifications are insufficient • Education on GDM pathophysiology, risks, and management strategies • Empowering patients with self-care techniques • Glucose tolerance testing post-delivery • Long-term follow-up for type 2 diabetes prevention

Table 11 documents clinical risk factors exhibited by features in the data set.

**Table 11:** Mapping data set to clinical risk factors.

Feature	Correlation with GDM
Number of pregnancies	May increase risk of β-cell dysfunction
Plasma glucose concentration	Directly reflects degree of glucose intolerance
Diastolic BP	May indicate underlying vascular issues affecting placental function
Triceps skin fold thickness	Reflects overall adiposity, contributing to insulin resistance
2-h serum insulin	Indicates body's insulin response to glucose challenge
BMI	Correlates with insulin resistance and overall metabolic health
Age	Associated with decreased β-cell function and increased insulin resistance
Diabetes Pedigree Function	Reflects genetic predisposition to diabetes

### 6.6 Effective drugs with clinical trials

Although most of the clinical trials are performed on Type-2 diabetes, the drugs in Table 12 are quoted to be safe for gestating mothers too [48].

**Table 12:** Drugs with Clinical trials for GDM management.

Ayurvedic Treatment	Dosage	Duration	Study Findings
Saptarangyadi Kashayam [49]	Ghanavati 200 mg of each, 5 Vati thrice a day,	2 Months	• Glycemic Control: Significant reductions in both fasting (12%, p < 0.01) and post-prandial (24%, p < 0.001) blood glucose levels. • Insulin Response: Statistically significant increase in post-prandial serum insulin levels (p < 0.05). • Long-term Efficacy: Significant reduction in HbA1c levels (p < 0.05) in selected patients. • Safety Profile: No significant adverse effects on renal function, as indicated by minimal changes in serum creatinine and urea levels.
Dadimadi Ghanam [50]	Dadimadi ghrita with dosage of10 ml	30 days	• Symptom Relief: 68.6% of subjects experienced over 50% relief, with 22.9% achieving 76–100% improvement. • Hemoglobin Improvement: 71.43% of subjects showed an increase of 1 gm% or more in hemoglobin levels. • Safety and Efficacy: Dadimadi ghrita improved anemia, digestion, and nourishment in pregnant women without side effects to mother or fetus.
Nishamalaki Churna [51]	6 gm Nisha-amalaki Churna two times a day before meals with lukewarm water	60-day treatment period and a follow-up period every 15 days.	• Fasting Blood Sugar: Mean FBS reduced significantly from 143.08 mg/dL to 129.39 mg/dL after treatment. • Post-Meal Blood Sugar: Mean PMBS decreased from 223.56 mg/dL to 209.26 mg/dL after treatment. • Safety and BMI: No adverse effects observed, and BMI slightly reduced from 26.06 to 25.86 after treatment.
Guduchi preparations or Tinospora cordifolia (TC) [52]	Performed on mice	21 days	• Showed the most promising results in reducing fasting blood glucose and improving insulin secretion • Lowered blood glucose levels and protected against pancreatic tissue damage • TC essential oil demonstrated hypoglycemic effects and improved beta cell function • All TC preparations increased serum insulin levels and insulin sensitivity • TC treatment lowered placental weight and increased litter size in mice • The study suggests TC preserves pancreatic beta cells and improves insulin production and function
Vijayasar extract [53]	• Vijayasar: 2–4 g/day • Tolbutamide (comparison drug): 0.75–1.5 g/day	• 36 weeks • 4 weekly clinic attendance for review and drug collection	• No significant difference in mean decrease of fasting or postprandial blood glucose between Vijayasar and tolbutamide groups (p = 0.2) • 86% of Vijayasar group and 94% of tolbutamide group achieved glycemic control • No significant changes in lipids and other laboratory parameters (p > 0.05) • No specific adverse effects related to Vijayasar • Vijayasar demonstrated comparable glycemic effect to tolbutamide in treatment-naive type 2 diabetes patients • Vijayasar was found to be an effective blood glucose-lowering agent without significant side effects

## 7 Conclusion

Gestational diabetes mellitus (GDM), also known as diabetes mellitus diagnosed during pregnancy, is a worldwide issue that is becoming important for public health. Negative perinatal outcomes, type 2 diabetes (T2D) in the mother later in life, and obesity in progeny are all significantly increased risks that are linked to GDM. Several experiments have been carried out to try to find the answer to the research question that is presented in this article. These tests are being conducted to see if it is possible to correctly diagnose diabetes in pregnant women using machine learning algorithms. Furthermore, the most important feature set rank-wise for GDM is developed through feature selection and importance. The quality of the dataset was also improved through imputation. Further along, these lines research can incorporate optimization algorithms to find best performing parameters for the elements in the architecture.

It is important to recognize the significant restrictions that apply to our research. First off, the location or region where the dataset was gathered is not explicitly stated as the gathered open source dataset was not region-specific. The generalizability of our findings may be impacted by this lack of geographical specificity. Furthermore, no real patients have been used to test the method. A pilot research or retrospective study utilizing a new intelligent algorithm based on symptoms and diagnosis would test the algorithm's effectiveness in a clinical environment and offer useful insights. These actions are essential for evaluating the possible significance and real-world relevance of our findings.

The lack of adequate data set is a major concern in all healthcare research owing to confidentiality issues. Techniques to effectively enhance the learning curve of algorithms under such conditions are also a possible research direction. The use and deployment of technology like AI, ML, and cloud in rural areas can effectively automate the process in such areas [54,55]. The available literature can be reviewed to draw the conclusion that Ayurvedic treatment doctrines (diet, yoga, herbs) can successfully manage gestational diabetes and ensure a healthy pregnancy for both the mother and the fetus. Nonetheless, a significant obstacle to the integration of herbal medicine into contemporary medical procedures is the absence of empirical and scientific data proving its efficacy and safety. Clinical research on herbal medicines is vital, as is the development of simple bioassays for biological standardised operations, evaluation of their pharmacological and toxicological effects, and development of many animal models for detrimental and efficacy assessment [55].

## Funding sources

The study did not receive financial support from public, private, or non-profit organizations.

## Author contributions

**NPS:** Methodology/Study design, Validation, Formal analysis, Writing – original draft, Writing – review and editing, Visualization. **JY:** Conceptualization**,** Validation, Formal analysis, Writing – review and editing. **SD:** Implementation, Validation. **VH:** Validation, Writing – review and editing. **MKV:** Supervision.

## Declaration of generative AI in scientific writing

No LLMs were used for drafting this manuscript.

## **Conflict of** interest

The authors declare that they have no known competing financial interests or personal relationships that could have appeared to influence the work reported in this paper.

## References

[1] Pranto B., Mehnaz S.M., Mahid E.B., Sadman I.M., Rahman A., Momen S. (2020). Evaluating machine learning methods for predicting diabetes among female patients in Bangladesh. Information.

[2] Adigun O., Okikiola F., Yekini N., Babatunde R. (2022). Classification of diabetes types using machine learning. Int J Adv Comput Sci Appl.

[3] Sharma H., Chandola H.M. (2011). Prameha in ayurveda: correlation with obesity, metabolic syndrome, and diabetes mellitus. Part 1–etiology, classification, and pathogenesis. J Alternative Compl Med.

[4] Kc K., Shakya S., Zhang H. (2015). Gestational diabetes mellitus and macrosomia: a literature review. Ann Nutr Metabol.

[5] Kim W., Park S.K., Kim Y.L. (2021). Fetal abdominal obesity detected at 24 to 28 weeks of gestation persists until delivery despite management of gestational diabetes mellitus. Diabetes Metab J.

[6] Sharma V. (2020). http://www.diva-portal.org/smash/get/diva2:1468839/FULLTEXT01.pdf.

[7] Xing J., Dong K., Liu X., Ma J., Yuan E., Zhang L., Fang Y. (2024). Enhancing gestational diabetes mellitus risk assessment and treatment through GDMPredictor: a machine learning approach. J Endocrinol Invest.

[8] Kang B.S., Lee S.U., Hong S. (2023). Prediction of gestational diabetes mellitus in Asian women using machine learning algorithms. Sci Rep.

[9] Liao L.D., Ferrara A., Greenberg M.B. (2022). Development and validation of prediction models for gestational diabetes treatment modality using supervised machine learning: a population-based cohort study. BMC Med.

[10] Du Y., Rafferty A.R., McAuliffe F.M., Wei L., Mooney C. (2022). An explainable machine learning-based clinical decision support system for prediction of gestational diabetes mellitus. Sci Rep.

[11] Zhang Z., Yang L., Han W., Wu Y., Zhang L., Gao C., Jiang K., Liu Y., Wu H. (2022). Machine learning prediction models for gestational diabetes mellitus: meta-analysis. J Med Internet Res.

[12] Ali N., Khan W., Ahmad A., Masud M.M., Adam H., Ahmed L.A. (2022). Predictive modeling for the diagnosis of gestational diabetes mellitus using epidemiological data in the United Arab Emirates. Information.

[13] Wang N., Guo H., Jing Y., Song L., Chen H., Wang M., Gao L., Huang L., Song Y., Sun B., Cui W. (2022). Development and validation of risk prediction models for gestational diabetes mellitus using four different methods. Metabolites.

[14] Jader R., Aminifar S. (2022). Predictive model for diagnosis of gestational diabetes in the Kurdistan region by a combination of clustering and classification algorithms: an ensemble approach. Appl Comput Intell Soft Comput.

[15] Shankar R.S., Raju V.S., Murthy K.V., Ravibabu D. (2021). In2021 5th international conference on electronics, communication and aerospace technology (ICECA).

[16] Ye Y., Xiong Y., Zhou Q., Wu J., Li X., Xiao X. (2020). Comparison of machine learning methods and conventional logistic regressions for predicting gestational diabetes using routine clinical data: a retrospective cohort study. J Diabetes Res.

[17] Qiu H., Yu H.Y., Wang L.Y., Yao Q., Wu S.N., Yin C., Fu B., Zhu X.J., Zhang Y.L., Xing Y., Deng J. (2017). Electronic health record driven prediction for gestational diabetes mellitus in early pregnancy. Sci Rep.

[18] Nagaraj P., Deepalakshmi P., Mansour R.F., Almazroa A. (2021). Artificial flora algorithm-based feature selection with gradient boosted tree model for diabetes classification. Diabetes, Metab Syndrome Obes Targets Ther.

[19] Khanam J.J., Foo S.Y. (2021). A comparison of machine learning algorithms for diabetes prediction. Ict Express.

[20] Gnanadass I. (2020). Prediction of gestational diabetes by machine learning algorithms. IEEE Potentials.

[21] Jader R.F., Aminifar S., Abd M.H. (2022). Diabetes detection system by mixing supervised and unsupervised algorithms. J Stud Sci Eng.

[22] Sumathi A., Meganathan S. (2022). Ensemble classifier technique to predict gestational diabetes mellitus (GDM). Comput Syst Sci Eng.

[24] Amrutha B.S., Padmasaritha K. (2020). Ayurvedic management of gestational diabetes mellitus - a case study. J Ayurveda Integr Med Sci.

[25] Paradkar S.R. (2017). Role of modern diagnostic methods in ayurvedic diagnosis: concepts & prospects. Int J Res AYUSH Allied Syst.

[26] Gayathri Bhat N.V., Dr Deepthi G.B. (2021). A rationale approach to Gestational Diabetes Mellitus through Ayurveda - Case Series. J Ayurveda Integr Med Sci.

[27] Yang J., Clifton D., Hirst J.E., Kavvoura F.K., Farah G., Mackillop L. (2022). Machine learning-based risk stratification for gestational diabetes management. Sensors.

[28] Ahsan M.M., Luna S.A., Siddique Z. (2022). Machine-learning-based disease diagnosis: a comprehensive review. Healthcare (Basel, Switzerland).

[29] Ghaffar Nia N., Kaplanoglu E., Nasab A. (2023). Evaluation of artificial intelligence techniques in disease diagnosis and prediction. Discov Artif Intell.

[30] Mohite S.S., Gajare R., Khose N.B. (November 2020). Comparative study of treatment of gestational diabetes mellitus cited in various ayurvedic and modern research papers published in last 5 years. Int Ayurvedic Med J.

[31] Kulkarni M., Chamwad V. (2024). Conceptual study of Garbhini Prameha with special reference to change in lifestyle. Afr J Biol Sci.

[32] Gholap S., Kumbhare S. (2021). Conceptual study of the efficacy of lodhra with honey gel in upapluta yoniyapad (vaginal candidiasis in pregnancy). Int Ayurvedic Med J.

[33] Sharma P., Sumit N., Kumar Sahu A., Bakuni H., Kumar Padhar B., Rawat S. (2022). Role of Kleda dushyain prameha(diabetes mellitus)-A critical review. Int Res J Ayurveda Yoga.

[34] Mohite S., Gholap S., Pawar K.P., Kulkarni M.B., Gajare R. (2021). A comparative review of ayurvedic classical garbhini paricharya and modern science antenatal care. Int J Res Ayurveda Pharm.

[35] Chavan V.R., Jana P., N C. (2021). Role of garbhini paricharya in the prevention aspect of GDM. Int J Mod Pharm Res.

[36] Koppikar V.S. (2008). Garbhini paricharya (regimen for the pregnant woman). Ancient Sci Life.

[37] Nanal V.R. (2008). Food in pregnancy an Ayurvedic overview. Ancient Sci Life.

[38] Koppikar V.S. (2008). Garbhini paricharya (regimen for the pregnant woman). Ancient Sci Life.

[39] Rakhshani A., Nagarathna R., Mhaskar R., Mhaskar A., Thomas A., Gunasheela S. (2012). The effects of yoga in prevention of pregnancy complications in high-risk pregnancies: a randomized controlled trial. Prev Med.

[40] Patel DrN. (2021). Role of yoga during third trimester. International Research Journal of Ayurveda & Yoga.

[41] Balaji P.A., Varne S.R. (2017). Physiological effects of yoga asanas and pranayama on metabolic parameters, maternal, and fetal outcome in gestational diabetes. Natl J Physiol Pharm Pharmacol.

[42] Youngwanichsetha S., Phumdoung S., Ingkathawornwong T. (2014). The effects of mindfulness eating and yoga exercise on blood sugar levels of pregnant women with gestational diabetes mellitus. Appl Nurs Res.

[43] Nadholta P., Bali P., Singh A., Anand A. (2020). Potential benefits of Yoga in pregnancy-related complications during the COVID-19 pandemic and implications for working women. Work.

[44] Chandla A., Tomer R., Gupta R. (2017). Gestational diabetes mellitus management through ayurveda. World J Pharm Pharmaceut Sci.

[45] Zhang J., Ma S., Guo C., Long S., Wu S., Tan H. (2018). Research progress on etiology of gestational diabetes mellitus. Glob Health J.

[46] Plows J.F., Stanley J.L., Baker P.N., Reynolds C.M., Vickers M.H. (2018). The pathophysiology of gestational diabetes mellitus. Int J Mol Sci.

[47] Sharma A.K., Singh S., Singh H., Mahajan D., Kolli P., Mandadapu G., Kumar B., Kumar D., Kumar S., Jena M.K. (2022). Deep insight of the pathophysiology of gestational diabetes mellitus. Cells.

[48] Muñoz Balbontín Y., Stewart D., Shetty A., Fitton C.A., McLay J.S. (2019). Herbal medicinal product use during pregnancy and the postnatal period: a systematic review. Obstet Gynecol.

[49] Singh K.S., Chandola H., Kaur M., Ravishankar B. (2012). Evaluation of Saptarangyadi Ghanavati in the management of Apathyanimittaja Prameha w.s.r. to type-2 diabetes mellitus. Ayu.

[50] Arankalle P.S. (2021). Effect of dadimadi ghrita in garbhini pandu (anaemia in pregnancy). J Ayurveda Holist Med.

[51] Chhabra D.A., Kuchewar V., Joshi T. (2024). Comparative study of *Nishaamalaki* and metformin in obese patients of type 2 diabetes mellitus (*Madhumeha*): a study protocol. F1000Research.

[52] Rani R., Chitme H., Sharma A.K. (2023). Effect of *Tinospora cordifolia* on gestational diabetes mellitus and its complications. Women Health.

[53] Hariharan Ramar, Venkataraman S., Sunitha P., Rajalakshmi S., Samal K.C., Routray B.M., Jayakumar R.V., Baiju K., Satyavati G.V., Muthuswamy Vasantha, Gupta A.K., Kumar N.K., Gupte Mohan, Radhakrishna S., Jayabal P., Venkatarao T., Tamby P.A., Kumar B., Selvaraj Vanathi, Mathai A.K. (2005). Efficacy of vijayasar (Pterocarpus marsupium) in the treatment of newly diagnosed patients with type 2 diabetes mellitus: a flexible dose double-blind multicenter randomized controlled trial. Diabetol Croat.

[54] Chaki J., Ganesh S.T., Cidham S.K., Theertan S.A. (2022). Machine learning and artificial intelligence based Diabetes Mellitus detection and self-management: a systematic review. J King Saud Univ-Comput Inf Sci.

[55] Bremer A.A. (2018). Commentary: research gaps in gestational diabetes mellitus: executive summary of a national institute of diabetes and digestive and kidney diseases workshop. Front Endocrinol.

